# It takes two to tango: the role of tumor-associated macrophages in T cell-directed immune checkpoint blockade therapy

**DOI:** 10.3389/fimmu.2023.1183578

**Published:** 2023-06-09

**Authors:** Fadi Sheban

**Affiliations:** Department of Systems Immunology, Weizmann Institute of Science, Rehovot, Israel

**Keywords:** tumor-associated macrophages, immunotherapy, cancer, combination therapy, tumor microenvironment

## Abstract

Immunotherapy has revolutionized cancer care in the past decade. Treatment with immune checkpoint inhibitors has demonstrated promising clinical activity against tumors. However, only a subset of patients responds to these treatments, limiting their potential benefit. Efforts to understand, predict, and overcome the lack of response in patients, have thus far focused mainly on the tumor immunogenicity and the quantity and characteristics of tumor-infiltrating T cells, since these cells are the main effectors of immunotherapies. However, recent comprehensive analyses of the tumor microenvironment (TME) in the context of immune checkpoint blockade (ICB) therapy have revealed critical functions of other immune cells in the effective anti-tumor response, highlighting the need to account for complex cell-cell interaction and communication underlying clinical outputs. In this perspective, I discuss the current understanding of the crucial roles of tumor-associated macrophages (TAMs) in the success of T cell-directed immune checkpoint blockade therapies, as well as the present, and the future of clinical trials on combinatorial therapies targeting both cell types.

## Introduction

Cancer immunotherapy has emerged in the late 19^th^ century when Dr. William B Coley treated cancer patients with intratumoral injections of live pathogens (1). The mechanism by which Coley’s pathogens led to the eradication of cancer was not clear at the time. However, today we understand that they elicit a local immune response that fights cancerous cells incidentally (2). In 1976, Coley’s work was followed by the first report of successful cancer immunotherapy using Bacillus Calmette-Guerin (BCG) to treat bladder cancer which is still in use until this day (3). A very important milestone in cancer immunotherapy was the discovery of immune checkpoints (4). These checkpoints are negative regulators of the immune system and particularly T cell activation and are evolutionarily conserved to fine-tune the duration and extent of immune responses to ensure self-tolerance (5, 6). Two prominent examples of immune checkpoints are the Programmed cell Death 1 (PD-1) and Cytotoxic T Lymphocyte Antigen 4 (CTLA-4). These receptors are expressed by activated T cells (among other cell types) and act as natural brakes allowing T cells to exert their function in a timely manner that ensures effective protection from cancer cells and pathogens while preventing autoimmunity (7, 8) In the last decade, inhibitory molecules that target these checkpoints, namely ICB therapies, have been developed and successfully used for the treatment of various cancer types. The success of ICB molecules in cancer treatment eventually earned James P. Allison and Tasuku Honjo the 2018 Nobel Prize in Physiology or Medicine (9).

Despite the promising results of cancer ICB therapy, durable clinical responses are limited to a subset of individuals with specific cancer types (10–12). Therefore, tremendous efforts were made to understand and predict the lack of response in patients. One of the most prominent biomarkers used to predict ICB response in some cancer types is tumor immunogenicity, which is primarily determined by tumor mutational burden, genomic instability, and efficiency of antigen presentation (13–16). The stronger the immunogenicity of the tumor, the higher the chances for a patient to benefit from ICB treatment. Additional biomarkers include the tumor immunophenotype of patients, which is mainly characterized by T cell infiltration into the TME. Hot tumors (immune-inflamed) are defined by high infiltration of T cells, whereas cold tumors (immune-desert) lack or have low T cell percentages (17–19). Patients with hot tumors were shown to be more likely to have a beneficial clinical response to ICB. Moreover, high expression levels of molecules that are targeted by ICB therapies such as PD-1 ligand (PD-L1) within the TME are considered as a biomarker for positive treatment responses (20). However, these parameters are limited to certain types of cancers and are not perfectly accurate. For instance, some patients which are considered negative for PD-L1 expression can still benefit from anti-PD-1/L1 treatment (21–24). This emphasizes that we still do not fully understand the mechanism of action (MOA) of such therapies and the need for further research to improve ICB therapy outcomes.

Recently, several comprehensive analyses of the TME have shed the light on the involvement of different immune cells, besides the extensively studied T cells, in the MOA of different immunotherapies. Specifically, TAMs were described as critical in shaping the TME and being directly involved in the treatment outcomes of immunotherapies. In this perspective, I describe the recent advancements in the understanding of the roles of TAMs in shaping anti-tumor responses elicited by cancer ICB therapies. Further, I highlight the potential of combination therapies targeting TAMs together with T cell-directed ICB molecules and the ongoing and future clinical trials.

## Tumor-associated macrophages

TAMs are an essential component of the TME and are usually key tumor-promoting players in most solid cancers. By orchestrating immunosuppression, angiogenesis, tissue remodeling, and tumor cell proliferation they support tumor growth and the formation of metastasis (25–28). As a consequence, high TAMs infiltration in solid tumors is usually associated with poor prognosis and resistance to chemotherapy and immunotherapy such as ICB therapies (29–37) Historically, macrophages have been divided into two functional phenotypes, referred to as M1 and M2 (38). This dichotomic model is based on the stimulation used to activate macrophages *in vitro*. M1 polarized macrophages are associated with inflammatory and anti-tumor activities, whereas M2 polarized macrophages are associated with resolution of inflammation and pro-tumor activities. However, *in vivo* in general, and specifically in the context of cancer, the phenotypes of macrophages are much more complicated. Single-cell analyses of human and murine TAMs have revealed several TAMs phenotypes that can even co-exist, indicating a more complex scenario beyond the simplistic M1/M2 classification (39, 40).

## The involvement of TAMs in responses to ICB therapies

Manipulation or depletion of TAMs in the TME can potentiate several immunotherapeutic strategies, including ICB, CAR-T cell therapies, and tumor vaccination (25, 41–43). This demonstrates the paramount role of TAMs in shaping treatment responses to immunotherapies. In the past decade, utilizing multi-omics techniques, several research groups have dissected the different functions of TAMs in the context of immunotherapy. In the settings of ICB therapy, TAMs have been shown to play critical expected, and unexpected roles in the effectiveness of various therapeutic molecules. Understanding these roles would help with finding novel biomarkers for response, defining potential therapeutic targets expressed by TAMs, and developing combinatorial therapeutic approaches to enhance the efficacy of the existing ICB therapies.

### TAMs as primary expressors of immune checkpoint ligands in the TME

**Figure 1:**
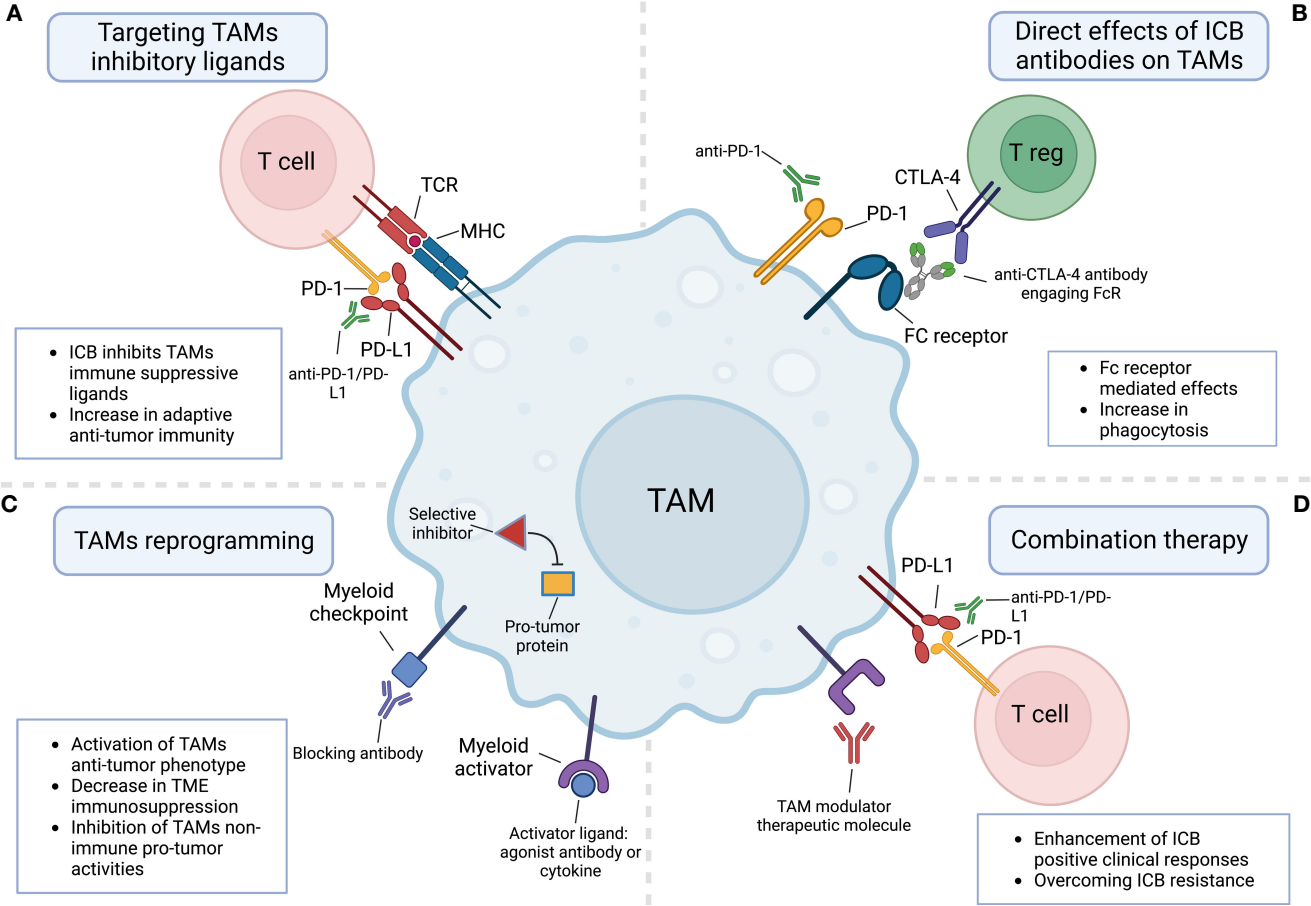
The involvement of tumor-associated macrophages in responses to immune checkpoint blockade therapies. **(A)** ICB molecules block TAMs cell surface immunosuppressive checkpoint ligands such as PD-L1 from interacting with their counterpart receptors on T cells. **(B)** TAMs directly respond to ICB molecules either by a direct interaction with the targeted receptor or through interaction with FC receptors expressed by TAMs. **(C)** TAMs can be reprogrammed by targeting their myeloid checkpoints receptors and pathways or by engaging their activating receptors. **(D)** Combination therapy combining ICB molecules and TAMs modulator therapeutic molecules boosts ICB clinical positive responses and overcomes resistance. Created with BioRender.com.

The foremost goal of ICB therapy is to unleash the cytotoxic activity of T cells which is limited by the immunosuppressive nature of the TME. TAMs are key players in suppressing adaptive anti-tumor immune responses (29, 44). Among the various mechanisms which TAMs apply to attenuate T cell activities, is the high expression of checkpoint ligands such as CD80, CD86, PD-L1, PD-L2, and CD155, etc. (**Figure 1A**) (45–47). The interaction of some of these ligands with their receptors on T cells was shown to downmodulate the amplitude of T cell activation and proliferation. For instance, PD-1-PD-L1 interaction drives inhibitory intracellular signals in T cells that eventually lead to T cell exhaustion (9). Assessment of PD-L1 expression in tumors is used as a diagnostic marker for anti-PD1 therapy in non-small cell lung cancer (NSCLC) and several other malignancies (17, 48). Both tumor cells and myeloid antigen-presenting cells (APCs) such as TAMs express PD-L1 in the TME. Yet, it was shown in animal models that PD-L1 on tumor cells was largely dispensable for the response to anti-PD1 therapy, whereas PD-L1 in host myeloid cells, was essential for this response (49). Further, it was shown that among myeloid cells, TAMs are the main source of PD-L1 in the TME. Depletion of PD-L1 in TAMs resulted in a reduction in tumor growth compared to control group, yet these effects were much stronger when depleted in dendritic cells (50). Interestingly, besides these extensively studied effects of the immune checkpoint-ligand interactions, it was shown that TAMs can also trap T cells by forming long-lasting interactions and impede them from reaching tumor cells (51). Importantly, the inability of T cells to infiltrate tumors is considered an important mechanism of resistance to ICB (17). Likewise, FasL^+^ TAMs promote liver metastases by inducing apoptosis of Fas^+^CD8^+^ T cells through Fas-FasL interaction, leading to an immune-desert TME (52).

More immune checkpoints ligands expressed by macrophages are being discovered and assessed for their ability to suppress T cell responses and thus their potential use as targets for ICB. V-domain Ig suppressor of T cell activation (VISTA) is a novel distinct immunoglobulin inhibitory ligand whose extracellular domain bears homology to the B7 family ligand PD-L1 (53, 54). By interacting with the adhesion and co-inhibitory receptor P-selectin glycoprotein ligand-1 (PSGL-1) on T cells, VISTA suppresses T cells selectively at acidic pH such as that found in TME (54, 55). Hence, VISTA-PSGL-1 axis is considered a new promising target for ICB and is now being assessed in clinical trials. Further research and screening for ligands expressed on TAMs that interfere with anti-tumor T cell responses will open the door for more discoveries and potential targets for ICB therapies.

### TAMs as effector cells of T cell-directed ICB therapy

ICB molecules that target PD-1-PD-L1 interaction have shown remarkable success in treating melanoma and NSCLC. Many monoclonal antibodies (mABs) targeting the PD-1-PD-L1 axis are FDA-approved and widely used to treat cancer patients. Yet, the MOA of these mABs is still not fully understood, limiting the advancement of novel therapeutic approaches and the improvement of the current clinical response rates. Early explanations of how these mABs work were based on the understanding that the two key player cells in the targeted axis are a T cell and another interacting cell expressing PD-1 and PD-L1 respectively. In this interaction, tumor or myeloid cells expressing PD-L1 suppress T cell responses by interacting with PD-1 on these cells.

Previously, it was reported that macrophages express PD-1 in the context of pathogen infection (56). In a more recent study, Gordon et al. aimed to assess whether macrophages might also express PD-1 in the context of cancer. Surprisingly, they showed that both human and mouse TAMs do express PD-1 in the TME. Interestingly, PD-1 expression levels negatively correlated with the phagocytic potency of TAMs against tumor cells, and *in vivo* blockade of PD-1 on TAMs improved their phagocytic activity, reduced tumor growth, and increased the survival of the mice (57). This study suggested that anti-PD-1 ICB therapy may also function through direct effects on TAMs and opened the door for further investigations. More recent studies confirmed this observation and showed in more detail how the deletion of PD-1 in TAMs induces anti-tumor immunity and suppresses tumor growth (58, 59). Interestingly, a recent study indicated that the functional effects of anti-PD-L1 therapy also are not only mediated by T cells. Instead, it seems that anti-PD-L1 treatment can reprogram TAMs into pro-inflammatory, antigen-presenting cells that help sustain and enhance effector CD8^+^ T cell activity (60, 61). However, it is not yet clear whether this is a direct or a secondary effect of anti-PD-L1 therapy.

These discoveries twisted the traditional view of the PD-1-PD-L1 axis as a T cell-specific immune checkpoint and brought light to a new role of TAMs as key effector cells in ICB therapy targeting the PD-1-PD-L1 axis.

As for anti-PD-1/PD-L1 therapy, the MOA of anti-CTLA-4 therapy is still under debate. The initial goal behind mABs targeting this molecule was to block inhibitory signals in activated effector T cells upregulating CTLA-4 and thereby unleashing their anti-tumor responses. However, more recently, several studies raised the possibility that these mABs might function by depleting or affecting the suppressive activity of regulatory T cells (Tregs) which constitutively express high levels of CTLA-4 (62). Moreover, it was shown that Fcγ receptor (FcγR)-dependent depletion of Tregs is crucial for the anti-tumor response elicited by anti-CTLA-4 (63). More recently, this (FcγR)-dependent depletion was shown to be accompanied with the remodeling of the myeloid compartment in the TME. Importantly, this immune remodeling was not driven solely by Treg depletion or CTLA-4 blockade, but mainly through FcγR engagement and downstream activation of monocytes and TAMs through type I interferon signaling (64). These findings indicated that FcγR engagement and TAMs remodeling are involved in successful anti-CTLA-4 treatment, emphasizing again the crucial emerging role of TAMs as direct effector cells in ICB therapies (**Figure 1B**).

### Reprogramming of TAMs overcomes resistance to ICB therapies

TAMs have been shown to regulate the therapeutic resistance mechanisms of different cancer therapies (33, 65–67). Therefore, whether TAMs regulate therapeutic resistance to ICB was an inevitable question. In one of the first studies assessing this, Zhu et al. showed that targeting TAMs by CSF-1R inhibition, enhanced the efficacy of either anti-PD1 or anti-CTLA4 therapies in an immunotherapy-resistant pancreatic cancer model (68). As of today, owing to several additional studies we understand that TAMs play a crucial role in resistance to ICB therapies (37). Thus, combining ICB with therapeutic agents impacting TAMs infiltration and/or activity has attracted particular attention and is being evaluated in clinical trials.

Even though TAMs are usually promoting tumor growth, and their infiltration is correlated with negative outcomes, eliminating them in the TME by blocking their recruitment or depleting them might be not the optimal solution to augment ICB efficacy (25). Instead, reprogramming them into anti-tumor TAMs would exploit their beneficial abilities to fight tumors such as activating T cells (rather than suppressing them), mediating direct cytotoxic tumor killing, and phagocytosis of dying tumor cells (**Figure 1C**). There are two strategies to functionally reprogram TAMs from pro-tumor into anti-tumor phenotype. The first one is by activating them towards an M1-like phenotype using receptors sensing pathogenic molecules and stimulation with inflammatory cytokines. The second strategy is by modulating their myeloid checkpoints and negative regulators. One prominent activator reported to repolarize TAMs and other myeloid cells is the CD40 receptor which is a member of the TNF receptor family expressed by APCs. When activated by its ligand CD40L, it triggers the production of anti-tumor cytokines and factors such as TNF and reactive oxygen species. Targeting CD40 by agonistic mABs powerfully enhanced responses of both anti-PD1 and anti-CTLA4 therapies in an immunotherapy-resistant pancreatic cancer model (69). Other macrophage activators include engagers of Toll-like receptors (TLRs), interferon receptors (IFNR), stimulator of interferon genes (STING), and FC receptors (25).

The physiological role of myeloid checkpoints and negative regulators of macrophage polarization is the protection of tissues from excessive inflammation and damage. Hijacking of such regulators by tumor cells results in immunosuppressive and tumor-promoting TAMs. One example of a frequently hijacked regulator is the CD47 “don’t eat me” signal (70). This protein is expressed on normal cells and interacts with SIRPa which is found on professional phagocytes to inhibit them from phagocytizing host cells. Tumor cells overexpress CD47 in many cancers and by this, they avoid their removal by TAMs and other phagocytes. Treatment with anti-CD47 or anti-SIRPa antibodies increased phagocytosis of cancer cells by TAMs and resulted in increased priming of CD8^+^ T to exhibit cytotoxic functions (71, 72). Other phagocytosis inhibitory receptor-ligand pairs include LILRB1–HLA1, SIGLEC10–CD24, and PD1–PDL1 (73, 74).

Scavenger receptors are highly expressed on TAMs and are associated with an immunosuppressive phenotype. Targeting scavenger receptors is a promising approach to reprogramming TAMs. For instance, the engagement of mannose receptor 1 (CD206) by a selective peptide (RP-192) that changes the receptor conformation, reprogrammed TAMs into an M1-like phenotype. Importantly, the combination of RP-192 and anti-PD-L1 therapies allowed to overcome ICB resistance in a pancreatic cancer model not known to respond to single agent of anti-PD-1/PD-L1 therapy (75). Macrophage receptor with collagenous structure (MARCO), macrophage scavenger receptor 1 (MSR1), and CLEVER-1 are other examples of scavenger receptors that mediate TAM reprogramming upon their targeting and are promising candidates for combination therapies with ICB (76–78). Recently, single-cell analysis of suppressive myeloid cells in tumors identified triggering receptor expressed on myeloid cells 2 (TREM2) to be a novel immunosuppressive gene expressed by myeloid subsets and especially TAMs in the TME (79, 80). Importantly, the modulation of the TREM2 pathway using blocking antibody remodeled the tumor myeloid landscape and enhanced anti-PD-1 therapy in a responsive tumor model (80, 81).

TAMs can be reprogrammed not only by agonizing or antagonizing their surface receptors but also by targeting intracellular proteins and pathways that are involved in the regulation of suppressive programs. Macrophage PI3Kγ was shown to control a critical switch between immune stimulation and suppression during inflammation and cancer, making it an ideal candidate for clinical purposes (82). Pharmacological inhibition of PI3Kγ resulted in efficient reprogramming of TAMs which synergized with anti-PD-1 treatments to reduce tumor growth. Another example is the epigenetic reprogramming of TAMs by inhibiting class IIa histone deacetylase (HDAC) (83). The inhibitor of HDAC, TMP195, was shown to alter the TME and reduce tumor growth and pulmonary metastases by modulating the phenotype of TAMs. Furthermore, combining TMP195 with anti-PD1 blockade significantly enhanced tumor size reduction in an otherwise resistant tumor model. Lastly, a recent report showed that the N6-methyladenosine reader YTHDF2 regulates the anti-tumor functions of TAMs (84). Ablation of YTHDF2 in TAMs suppressed tumor growth by reprogramming TAMs toward an antitumoral phenotype and increasing their antigen cross-presentation abilities, which in turn enhanced CD8^+^ T cell-mediated anti-tumor immune responses. In line with this observation, the ablation of YTHDF2 enhanced the efficacy of anti-PD-L1 therapy.

Standard of care cancer treatment such as chemotherapy was also shown to affect TAMs phenotype (66, 85, 86). Paclitaxel is a common chemotherapeutic drug used to treat various types of solid tumors. Recent studies have revealed that Paclitaxel not only inhibits cancer growth through its traditional cell-cycle arrest mechanism, but it can also reprogram TAMs in a TLR4-dependent manner, resulting in an enhanced immune response against the tumor (87, 88). Thus, such immunostimulatory chemotherapies appear to be a promising combination partners of ICB therapies, although further research is needed to optimize such treatment regimens (89).

Together, the different examples presented here demonstrate the paramount importance of TAMs modulation in gaining a successful clinical response to different T-cell-mediated immunotherapies. Future studies that will discover additional suppressive genes and pathways, will broaden the arsenal of possible clinical TAMs targets, and increase the chances for successful ICB combinatorial treatment in patients.

### TAMs modulators in combination with ICB therapies - present and future clinical trials

The sum of the mentioned discoveries encouraged clinical trials combining TAM modulators with different ICB therapeutic agents (**Figure 1D**). Most of the selected ongoing clinical trials (**Table 1**) evaluate the combination of a novel TAM modulator agent with an existing ICB therapy. The more data and clinical parameters that will be collected from these clinical trials the deeper our understanding will be of such combination therapy approaches in humans. For instance, single-cell profiling of tumor biopsies before treatment (if possible) would enable us to correlate TAMs infiltration and expression of specific markers by TAMs with clinical outcomes. This would allow the acquisition of more detailed insights on the patients that are more likely to respond to combination therapy and help to design future data-driven clinical trials. Moreover, as discussed earlier, the binding of therapeutic antibodies to macrophage Fc receptors is critical for their clinical output (90). Thus, profiling of Fc receptors on myeloid cells in the TME would boost our knowledge of the correlation between the Fc receptors landscape and clinical responses. Additionally, understanding these basic concepts will critically improve the way we design antibody-based drugs in the future.

**Table 1:** Selected clinical trials of combination therapies targeting tumor-associated macrophages together with immune checkpoint blockade agents.

TAM targeting agent	Combination ICB agent	Cancer type	Clinical Trial
TLRs activation agonist			
TLR3 agonist	anti-PD-L1	Biopsy-accessible tumors	NCT02643303
	anti-CTLA-4		
	anti-PD-1	Melanoma	NCT04570332
TLR7 agonist	anti-PD-1	Her2^+^ solid tumors	NCT04278144
TLR7/8 agonist	anti-PD-1	Metastatic solid tumors	NCT04799054
TLR9 agonist	anti-PD-1	Melanoma	NCT04401995
CD40 agonists			
	anti-PD-1	Several tumor types	NCT02376699
	anti-PD-1	Melanoma and renal carcinoma	NCT04495257
	anti-CTLA-4		
	anti-PD-1	Solid tumors	NCT05165433
	anti-PD-1	Pancreatic cancer	NCT03214250
	anti-PD-1	Metastatic melanoma	NCT02706353
SIRPa/CD47 blockers			
anti-CD47	anti-TIGIT	Urothelial Carcinoma	NCT03869190
	anti-PD-L1		
anti-SIRPa	anti-PD-1	Solid tumors	NCT03990233
CLEVER-1 blocker			
	anti-PD-1	NSCLC	NCT05171062
Trem2 inhibitor			
	anti-PD-1	Solid tumors	NCT04691375

The clinical interest in combination therapy is already high due to the resistance of certain cancer types and individuals to ICB monotherapy. If some of the current combination therapy clinical trials will prove their enhanced efficacy over monotherapies, this will further fuel the enthusiasm for combination therapies, and we will see much more clinical trials in the next years. With the ongoing advances in antibody design, future clinical trials will probably include more sophisticated antibodies. For instance, bi-specific antibodies that target a myeloid checkpoint while activating T-cells or engaging Fcγ receptors could be of high clinical potential. Finally, further basic research that involves large and well-designed perturbation screens will allow us to bring more TAM-specific targets to clinical trials.

## Data availability statement

The original contributions presented in the study are included in the article/supplementary material. Further inquiries can be directed to the corresponding author.

## Author contributions

The author confirms being the sole contributor of this work and has approved it for publication.
